# The dance of hair – toward a more powerful performance

**DOI:** 10.3389/fpsyg.2023.1214861

**Published:** 2023-08-22

**Authors:** Maja S. Vukadinović

**Affiliations:** School of Business, University of Novi Sad, Novi Sad, Serbia

**Keywords:** hair, dance, the use, technique, performance

## 1. Introduction

Ever since ancient times, hair has been associated with different interpretations, and there is a solid corpus of literature which has explored understanding, symbolism and attitudes toward hair in different cultures, relating them to anthropological, social, and psychological aspects (Leach, 1958; Synott, 1987; Gheerbrant and Chevalier, 1996; c.f. Barkai, 2016; Sherrow, 2023). For example, in distant past in many cultures long loose hair were characteristic of a warrior's dignity and strenght no matter if the warrior was man or woman (Roberts, 2010; Sherrow, 2023). In Victorian era, aesthetically capturing long loose hair was often associated with sexual liberty and women's sensuality (Ebbatson, 2011). On one side hair was a symbol of strength, beauty and sexuality. However there is evidence that long loose hair as well may be related with immorality or shame of a person who is not capable of controlling his or her instinctive nature (Synott, 1987; Barkai, 2016). In some cultures long loose hair may be an indicator of a person's marital status where women gave up their long hair when they got married (Bilu, 2006). Also, Thompson (2008) suggests that, for instance in Africa, hairstyles may indicate a person's marital status, age, religion, or ethnic identity. In addition to this Afro hair is often associated with slavery and the reason for discrimination against different communities such as black people (Patton, 2006; Thompson, 2008). Thus, as a social symbol hair conveys meanings about roles, status, or attitudes uniting within its connotation both bright and dark sides of its possible meaning (Bartlett, 1994; Gheerbrant and Chevalier, 1996).

As a subject of aesthetic experience and means of communication, long hair often appears as a highly symbolic metaphor in different domains of art. Some examples include the paintings *Vampire* by Edvard Munch (1895) and *Medusa* by Caravaggio (1597), the 1960s musical *Hair*, as well as Walt Disney's 3D computer-animated film *Tangled* (2010) based on the story of Rapunzel.

In the domain of dance as a form of art, hair can be used for different single or combined purposes such as aesthetic, symbolic or instrumental ones. Hairstyles typical for a particular dance form are important elements of the overall aesthetic experience. In classical ballet, a ballerina's hair is pulled up into a high or low bun (Wulff, 2020), while contemporary dancers may have loose hair depending on the character they are interpreting. Strict hair styling is a distinctive feature of ballroom dance (Uba, 2007), and the low bun was, until recently, an imperative in flamenco dance (Ruiz, 2007). Regarding the symbolical level, hair in dance performance could be used as a metaphor for depicting different ideas related to strength, sexuality, sensuality, creativity, freedom and their opposites (Barkai, 2016), as well as different states such as grieving and mourning characterized by dramatic waving of head with long hair (Briand, 2013). Concerning its instrumental purpose, as a part of a dancer's body, hair can serve as an expressive means of dance.

This article focuses on hair as a dancing instrument and discusses how its manipulation could be incorporated into dance techniques. Taking into account three main purposes of the use of hair (decorative, symbolic and instrumental) the reason why I focus on specific hairstyle – long loose hair that stretches along the entire back – lies in our interest to explore its manipulation as a part of dance technique. In addition, I also focus on women since until now in modern literature long loose hair has been discussed only in men who dance flamenco (Washabaugh, 2020).

## 2. Performance, dance technique and hairstyles

Dealing with the use of hair in dance performance, I set its instrumental purpose into the framework of dance technique and the rhythmicity each dance technique assumes. Regardless of the dance form, during a performance, the dancer rhythmically moves to the music synchronizing his or her expressive body movements to the beat, where the common component of music and dance is the rhythm (Jordan, 2011). Rhythm in dance refers to the temporal structuring of movement in space, and it is the basis for the spatiotemporal synchronization of movement (Thaut, 2007; Luck and Sloboda, 2009). The kind of dance movements that would be performed and rhythmically synchronized depends on the dance technique characteristic of each particular dance form. Siegel (1972) and McFee (1992) understand dance technique as a method of dancers' physical training to attain a certain degree of physical fitness and skills so that they can perform specific dance movements (McFee, 1992, p. 211). Dance technique is also determined by a systematic approach to the entire process of dance movement (Siegel, 1972, p. 106). Usually, it includes typical handwork, footwork, headwork, turns, poses, and how movements are combined.

On the one hand, there are dance forms with specific hairstyles that are not part of the technique but an attribute of a dancer's final appearance. Classical ballet, for example, with its specific hair styling – ballet bun – focuses on the technique of the body so that with each segment, perfect form and “the aesthetics of order” of classical ballet are emphasized. Thus, the hairstyle is at the service of the entire dance form, i.e. regardless of how long it is, collected in a bun, hair is not used in the context of the dance technique. Furthermore, a ponytail, as a hairstyle characteristic of Broadway or Ballroom dance and an aesthetic requirement, remains at the level of decoration. On the other hand, there are forms in which hairstyle tends to be used as a metaphor, as a dancing instrument or both. For instance, contemporary flamenco dance from the last decade exhibits a trend for dancers, not just female but male as well (Washabaugh, 2020), to perform dance movements wearing long, loose hair, which used to be merely an exception. Such inclinations could be a metaphor emphasizing strength, sensuality and passion which are some of the main attributes of flamenco dance (Gómez Muñoz, 2008). In contemporary dance, there are also pieces where long hair symbolizes freedom, for example, in the movie *Pina* (2011). Moreover, in these dance forms, loose hair is often used as an element of the head-moving technique. In contrast, it should be mentioned that in breakdance, hair restricts dancers when performing head-spinning techniques.

### 2.1. The practice and use of loose hair in dance performances

When considering hair as a part of the dance technique, I will focus on long, loose hair. Two main questions should be addressed: How is the manipulation of long loose hair practiced? How and where can it be applied in dance performance?

For the dancer, the kinesthetic-vestibular system has an important role. It includes proprioception, which contains muscle and joint sensitivity (limb movement), while vestibular sensitivity refers to body and head orientation in space (Montero, 2006, 2012; Proske, 2006; Tuthill and Azim, 2018; Beck et al., 2020). Research dealing with the role of visual and proprioceptive information shows that dancers rely on the sense of vision when learning and practicing a specific step or movement sequence in front of a mirror (Dearborn and Ross, 2006; Shabbott, 2010). At the same time, they practice a specific movement in classes until they achieve proprioceptive integration of information and bodily representation of movement (Jola and Davis, 2011). Furthermore, different studies have shown that dancers rely more on vestibular and proprioceptive cues than on vision when determining body position and orientation (Golomer and Dupui, 2000; Jola and Davis, 2011; Beck et al., 2020). In performance, proprioception and the sense of vision function in a relationship of interdependence (Montero, 2006). The flow of information between the dancer's proprioceptive sensitivity and visual aesthetic sensitivity occurs in both directions (Jola and Davis, 2011; Montero, 2012). Thus, practice and use of the technique of hair manipulation are based on proprioception and vestibular sensitivity, head movement and sense of vision. Moreover, they include skin sensation as well, since long, loose hair falls on neck and shoulders.

With all this in mind, incorporating hair movement into dance technique will first assume awareness of its weight, volume and length through the sensations perceived in the skin and visual sensory modality. Secondly, it will implicate the exploration of how hair “behaves” during different movements, such as whether it turns, falls or jumps, as well as, where it tends to fall and end during frontally or diagonally positioned movements. Moreover, it will mean discovering the specific velocity at which hair moves and synchronizing it with body movement. Finally, it will require exercises of synchronization of hair manipulation, body movement and rhythm to which dance is performed, i.e. the sensorimotor coupling of dance and music.

Another question relates to the potential application of the technique of manipulating long, loose hair. I will mention a few interesting possibilities. One is related to the enhancement of the dynamics of dance. The dynamics of dance entails the speed of shifting certain dance movements and implies the change of fast and slow movements as well as pauses (Hagendoorn, 2008). Thus, training in hair manipulation could be applied to enhance dance dynamics, adding to the visual experience impression of acceleration or bringing additional liveliness and vibrancy to the movement. Moreover, it could contribute to the experience of keeping the pause, i.e. movement of stillness in dance, alive. Since the pause in dance is also related to its dynamics, a powerful use of loose hair manipulation could be applied in accentuating the pause between two dance sequences where the body remains still.

A further possibility of using hair in dance is related to situations in which the dancer's breath moves it. It has been shown that people with a prominent openness personality trait enjoy hearing the dancer breathe while performing without music, while those with a low score on this personality trait perceive the dancer's breathing as disturbing, eerie, uncomfortable and awkward (Jola et al., 2014). Considering the aesthetic impact and enjoyment that some spectators draw from dancers' breathing, long, loose hair can be intentionally used to emphasize it. For example, hair that falls over the dancer's face allows the breathing to be 'seen' but not heard. That way, the type of audience who dislike hearing the dancer's breath can also enjoy it and the aesthetical impact of dance can be achieved across all types of spectators, both those who like hearing the dancers' breath and those who do not.

Long loose hair as a part of the dance technique can also be considered in relation to the phenomenon of “visual capture”. Even in other art forms long, loose hair has always been visually captive, provoking aesthetic experience and spectator's arousal (Sherrow, 2023). Jordan (2011) explains that “visual capture” occurs when the perception of music is influenced by the movement so that if the musical sequence is heard alone, it may be barely perceptible. Choreographers may use turns, jumps or a combination of the two to create visually captive movements in dance performances. These movements, combined with the technique of manipulation of long loose hair, may put a more powerful accent on the beat or a certain moment in the music.

## 3. Discussion

The use of long loose hair and its incorporation into dance technique raise a number of key issues which I have elaborated upon above. The first one relates to getting to know the “instrument” (i.e., hair weight, volume and length as well as its “behavior” during different movements). The second one relates to the training of how to use long loose hair to express and enhance the dynamics of dance via specific movements or via breathing. The last point relates to the manipulation of long loose hair to evoke the phenomenon of “visual capture”.

However, the hair is just one element of the entire dance performance which requires a complex organization and whose final outcome and aesthetical impact depend on various factors related to the characteristics of dancers, their professionalism, level of training, costumes and make-up used, scenography, lighting, size of the stage, music etc. (Glass, 2005; Stevens, 2005; Jaeger, 2009; Geukes et al., 2023).

Although this article deals with only one aspect of dance performance, it has offered essential insight into different levels and possibilities regarding the use of long loose hair. I consider this to be important because until now, the role of hair in dance has been approached from the perspective of aesthetics (Ruiz, 2007; Uba, 2007; Washabaugh, 2020; Wulff, 2020; Sherrow, 2023), dance therapy (Barkai, 2016) or health injuries (Monselise et al., 2011; Hall et al., 2022; Wanke et al., 2022). However, to the best of our knowledge, the manipulation of hair as a part of the dance technique and its possible application in dance performance have not been fully addressed yet. Although this article provides only an overview of this topic, the incorporation of manipulation and use of hair within dance techniques with an aim to empower dance performances should be discussed and elaborated thoroughly. Future studies should be enriched by also exploring the choreographer's experience. Moreover, the investigation of its practical applications by scientist as well as by choreographers and dancers within each dance form would be fruitful and recommendable. Since the mentioned studies focused mostly on white people and those identifying as women, both performance art and science would benefit from future research dealing with application and use of long loose hair across sexes, genders, and cultures. In addition, questions connected to the possible relationship between a dancer's age, their social status, and their “body identity” (Langdon and Petracca, 2010) in relation to the use of long loose hair would be important to address to achieve more inclusive perspectives and gain greater insight into this key topic in the field of dance performance.

## Author contributions

The author confirms being the sole contributor of this work and has approved it for publication.
